# Tracking Measles and Rubella Elimination Progress—World Health Organization African Region, 2022–2023

**DOI:** 10.3390/vaccines12080949

**Published:** 2024-08-22

**Authors:** Balcha G. Masresha, Charles Shey Wiysonge, Reggis Katsande, Patrick Michael O’Connor, Emmaculate Lebo, Robert T. Perry

**Affiliations:** 1 WHO Regional Office for Africa, Brazzaville P.O. Box 06, Congo; sheyc@who.int (C.S.W.);; 2 WHO Headquarters, 1211 Geneva, Switzerland; oconnorp@who.int; 3 US Centers for Disease Control, Atlanta, GA 30333, USArmp9@cdc.gov (R.T.P.)

**Keywords:** measles, rubella, elimination, Africa, vaccination, coverage, surveillance

## Abstract

Measles or rubella elimination is verified when endemic transmission of the corresponding virus has been absent for over 36 months in a defined area, in the presence of a well-performing surveillance system. This report updates the progress by 47 countries in the WHO African Region towards the goal of attaining verification of measles and rubella elimination in at least 80% of the countries of the region by 2030. We reviewed the WHO-UNICEF vaccination coverage estimates for the first and second doses of measles- and measles-rubella-containing vaccines, as well as the available coverage data for measles supplementary immunization activities, during 2022–2023. We also reviewed the measles-surveillance performance and analyzed the epidemiological trends of measles and rubella as reported in the case-based surveillance database. The WHO-UNICEF estimates of first measles vaccine dose (MCV1) and second measles vaccine dose (MCV2) coverage for the African Region for 2022 were 69% and 45%, respectively. Rubella-containing vaccines have been introduced in the routine immunization program in 32 of 47 (68%) countries as of the end of 2022, with no introductions during 2023. In 2022 and 2023, a total of 144,767,764 children were vaccinated in the region with measles or MR vaccines in 24 countries through 32 mass vaccination campaigns. The administrative coverage target of 95% was reached in only 15 (49%) of the 32 vaccination campaigns. In 2023, a total of 125,957 suspected cases of measles were reported through the case-based surveillance system, and 73,625 cases (58%) were confirmed to be measles, either by laboratory testing, by epidemiological linkage, or based on clinical compatibility. A total of 4805 confirmed rubella cases were reported, though this total represents substantial under-ascertainment. The regional incidence of measles was 60.3 cases per million population. Twenty-six countries (55%) met the targets for the two principal surveillance system performance-monitoring indicators. No country in the region has attained the verification of measles or rubella elimination as of the end of 2023. Addressing systemic problems with routine immunization and using tailored approaches to reach unvaccinated children can contribute to progress towards measles and rubella elimination. In addition, periodic and timely high-quality preventive SIAs remain a critical programmatic strategy to reach unvaccinated children.

## 1. Introduction

In 2011, the 46 member states (South Sudan joined the WHO African Region in 2012) in the WHO African Region adopted the goal of measles elimination by 2020, with programmatic targets including ≥95% coverage with two doses of measles-containing vaccine (MCV) at national and district levels through routine immunization or supplementary immunization activities (SIAs); a confirmed annual measles incidence of less than one case per million population; and case-based measles-rubella surveillance performance that meets the performance targets [1]. Countries would be verified as having eliminated measles or rubella when endemic transmission of the corresponding virus has been absent for over 36 months, in the presence of a well-performing surveillance system [2]. However, by the end of 2020, none of the countries in the region had been verified for measles or rubella elimination. 

In 2021, as part of the strategic framework of implementation of the Immunization Agenda 2030 (IA2030), the regional goal was revised to attaining verification of measles and rubella elimination in at least 80% of the countries of the region by 2030 [3].

In 2020 and 2021, during the COVID-19 pandemic, many countries in the African Region and beyond experienced a decline in immunization coverage and surveillance performance [4]. In addition, scheduled introduction of rubella vaccine and second dose of measles or measles-rubella (MR) vaccines were delayed in some countries, and scheduled measles and MR campaigns were postponed, leading to increasing immunity gaps among vulnerable populations [5].

Despite the efforts to strengthen the routine immunization, the regional coverage with the first MCV dose has remained stagnant over the last decade [6].

This report describes progress towards the regional measles- and rubella-elimination goals, updating an earlier report covering measles-elimination through 2021 [7].

## 2. Methods

We reviewed the WHO-UNICEF vaccination coverage estimates for the first and second doses of MCV, which were available at the time of this analysis only for the year 2022, as well as the available coverage data from 2022 and 2023 for supplementary immunization activities utilizing measles- and measles-rubella-containing vaccines. We also reviewed measles-surveillance performance and analyzed the epidemiological trends of measles as reported in the case-based surveillance database. In addition, we reviewed country progress to establish national verification committees and reports of meetings of the Regional Verification Commission to assess progress in countries towards achieving measles or rubella verification.

WHO and UNICEF generate country-specific annual estimates of vaccination coverage for all antigens, including for the first and second measles-containing vaccine doses (MCV1 and MCV2, respectively) delivered through routine immunization services [8].

Countries are recommended to conduct measles or measles-rubella SIAs (implemented through a mass vaccination campaign effort) before the estimated number of children under five years of age and susceptible to measles approaches the size of one birth cohort. Countries are requested to report the timing, target age range and geographic areas targeted (if not nationwide), and administrative coverage, defined as the number of doses delivered during the campaign divided by the estimated target population. Coverage should also be documented through a high-quality post-campaign coverage survey [9].

All 47 countries in the African Region implement case-based measles surveillance and are expected to provide data on suspected and confirmed cases to the WHO on a weekly basis. Suspected measles cases are investigated at district level, with the investigation including relevant epidemiological information using a standard case investigation form and the collection of serum specimens for serological testing [10]. Testing is done in one of the 52 serological laboratories in the 43 countries of the regional measles laboratory network. Laboratories are regularly accredited to meet performance standards by the WHO Global Measles and Rubella Laboratory Network (GMRLN) [11].

Case-based surveillance performance is regularly monitored using standard performance indicators against annual targets such as the number of discarded cases of non-measles febrile rash illness per 100,000 population annually (target: two or more per year), and the proportion of districts reporting at least one suspected case with the collection of a blood specimen (target: ≥80% of districts per year) [10].

A confirmed case of measles is a suspected case testing positive for measles-specific IgM antibody who had not received measles vaccination within 30 days before the specimen collection, or a suspected case with epidemiological linkage to a laboratory-confirmed case of measles during a confirmed outbreak period, or a suspected case clinically compatible but not investigated with a lab specimen or linked to an outbreak [10].

Some national serological laboratories conduct rubella IgM testing for all measles seronegative specimens, while others run the tests in parallel. Therefore, the measles-surveillance system also generates data on the epidemiology of rubella in the countries. 

In addition to serum specimens for IgM testing, surveillance officers are expected to collect throat swab specimens from patients with acute cases of measles and rubella to send to designated virological laboratories to identify and characterize the circulating viral strains.

For some countries, we also reviewed data on measles cases from aggregate reporting systems. Reports give the number of reported suspected measles cases weekly and by health district. These countries, generally with large disease burdens, officially report aggregate numbers of suspected measles cases to WHO and UNICEF during the annual joint reporting process.

## 3. Results

### 3.1. Coverage through Routine Immunization

The WHO UNICEF estimate of MCV1 coverage for the African Region for 2022 was 69% (increasing from 68% in 2021), while the estimate of MCV2 coverage was 45% (increasing from 41% in 2021). Five countries reported ≥95% MCV1 coverage in 2022 (Cabo Verde, Ghana, Mauritius, Rwanda, and Seychelles). Comparing the 2022 MCV1 coverage estimates against those for 2021, 18 countries documented increased coverage. The increase was >5 percentage points in six countries, Guinea Bissau, Liberia, Mauritania, Mauritius, Rwanda, and Tanzania, with Liberia increasing by 21 points, from 58% in 2021 to 79% in 2022. On the other hand, 13 countries had lower MCV1 coverage levels in 2022. The coverage decline was more than five percentage points in Botswana, Gabon, Lesotho, Malawi, Niger, and Senegal, with the biggest decline of 15% points documented in Niger. MCV1 coverage in 2022 remained the same as the estimates for 2021 in 16 countries (Table 1).

**Figure 1:**
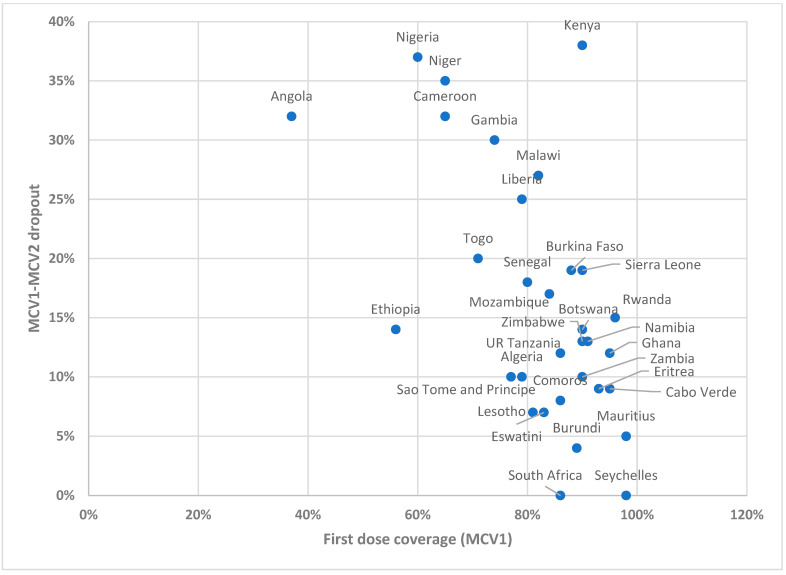
Measles first dose coverage and drop-out rates by country. African Region, 2022.

The trends indicate an increase in MCV2 coverage since 2012, with an increasing number of countries introducing a second dose measles vaccine in the past decade. Out of the 41 countries which had WHO-UNICEF coverage estimates of MCV2 in 2022, only Seychelles attained >95% coverage. Amongst the countries that had had MCV2 in their routine immunization program for more than 3 years, the drop-out rate between MCV1 and MCV2 is 10% or more in 23 countries, while the dropout rate is 20% or more in 9 countries (Figure 1).

Rubella-containing vaccines (RCVs) had been introduced in the routine immunization program in 32 (68%) of 47 countries as of the end of 2022, with no country having introduced it in 2023. The remaining 15 countries include three countries not eligible for support from Gavi, the Vaccine Alliance (Equatorial Guinea, Gabon, and South Africa), and 12 Gavi-eligible countries that have yet to meet the requirements set by WHO of 80% measles vaccination coverage through SIAs or through the routine vaccination program before receiving Gavi support to introduce RCV (Central African Republic (CAR), Chad, Democratic Republic of Congo (DRC), Ethiopia, Guinea, Guinea Bissau, Liberia, Madagascar, Mali, Niger, Nigeria, and South Sudan). The WHO-UNICEF estimate of first dose rubella-containing vaccine coverage in 2022 was 36%, reflecting the large countries in the region not yet having introduced the vaccine.

As of December 2023, at least 12 countries had utilized measles-containing vaccines packaged in 5-dose or lesser dose vials for routine vaccination service. These are Botswana, Cabo Verde, Comoros, DRC, Equatorial Guinea, Eritrea, Eswatini, Lesotho, Mauritius, Niger, Seychelles, and Zimbabwe. 

### 3.2. Supplementary Immunization Activities (SIAs)

Countries in the African Region continue to implement periodic follow-up vaccination campaigns to reach unvaccinated children and close immunity gaps, especially in the youngest birth cohorts. These campaigns are scheduled at an interval based on the rate of accumulation of unprotected children within the under-five-years age group and based on the epidemiology of measles in the countries. In 2022 and 2023, a total of 24 countries implemented 32 preventive or outbreak-response vaccination campaigns, reaching a cumulative total of 144,763,470 children (Table 2).

The administrative coverage target of 95% was reached in 15 of these campaigns. Post-campaign coverage surveys were implemented after only 6 campaigns, and survey coverage showed that 95% coverage was not attained in any of the campaigns in the four countries where results are available (Madagascar, Malawi, Nigeria, and Zimbabwe). Official results are not yet available from the post-campaign surveys in Senegal or Ethiopia. 

### 3.3. Measles and Rubella Surveillance and Incidence

In 2022, a total 114,347 suspected cases of measles were reported in the African Region through the case-based surveillance system, of which 60,857 (53%) were confirmed by lab, epidemiological linkage, or clinical compatibility. The regional incidence of confirmed measles was 51.5 per million population. Nigeria, Ethiopia, Liberia, and Zimbabwe accounted for 37,815 (62%) of the confirmed measles cases. The highest incidence of confirmed measles was in Liberia (1162 cases per million population), followed by Gabon (489 cases per million) and Zimbabwe (289 cases per million), and Nigeria (103 cases per million). The annual detection rate of non-measles febrile rash illnesses (NMFRI) was 3.8 per 100,000 population, and 89% of districts reported at least one suspected measles case with a blood specimen. A total of 28 countries met the targets (NMFRI rate of at least 2 per 100,000 and at least 80% districts reporting) for these two performance indicators in 2022.

In 2023, a total 125,957 suspected cases of measles were reported through the case-based surveillance system. Blood specimens were collected from 77,998 cases (62%), of which 22,258 cases (29%) were confirmed to be IgM positive for measles. Of the cases without a specimen, 42,098 cases were confirmed by epidemiological linkage, and 9269 cases confirmed as clinically compatible measles. As of the end of March 2024, a total of 1163 cases reported in 2023 still had pending lab results or pending case classification in the surveillance database. 

In 2023, the largest number of confirmed measles cases was reported in Ethiopia (16,505 cases), Nigeria (12,245 cases), DRC (10,662 cases), and Cameroon (6174 cases). The regional incidence of measles was 60.3 cases per million population (22% increase from 2022), while the highest national incidence rates of confirmed measles were documented in Liberia (831.6 cases per million population), Gabon (480.4), Equatorial Guinea (268), Central African Republic (240), Cameroon (217.1) and Ethiopia (154.1). Only 3 countries (Cabo Verde, Algeria, Seychelles) attained incidence of <1 per million population, while 6 countries reported measles incidence of 1–4.9 per million population, and 5 countries reported incidence of 5–9.9 per million (Table 3).

According to the case-based surveillance data, the annual detection rate of non-measles febrile rash illness for 2023 was 4.2 per 100,000 at the regional level with 34 (72%) countries meeting the target of at least 2 per 100,000. At the regional level, 87% of the districts investigated at least one suspected case with a blood specimen in 2023. Thirty-four (72%) countries met the target of at least 80% districts investigating one or more suspected cases with a blood specimen. Twenty-six countries (55%) met the targets for the two principal surveillance performance indicators in 2023, while three countries (6.4%) missed both targets.

Countries also receive weekly reports of suspected measles cases through aggregate reporting systems. Some countries have operational or other challenges that prevent them from reporting all suspected measles cases through the case-based surveillance system, such as DRC (311,500 suspected measles cases notified through aggregate reporting in 2023), Guinea (910 suspected cases in the aggregate reporting database for 2023), and South Sudan (7957 suspect cases in 2023). These countries are typically those with large outbreaks. 

A total of 4805 lab-confirmed rubella cases were reported in the region for 2023, of which 933 (19%) were from Ethiopia, 746 (16%) from Nigeria, 591 (12%) from DRC, 440 (9%) from Tanzania, and 389 (8%) from Madagascar. The regional incidence of laboratory-confirmed rubella was 4.0 cases per million population. The highest rubella incidence was reported in Guinea Bissau, at 74 per million population, with 155 lab-confirmed rubella cases (Table 3).

In 2022, seven countries documented the genotype of circulating measles viruses, with D8 genotype viruses detected in South Africa and Zimbabwe, while B3 genotype viruses were documented in Algeria, Benin, South Africa, Tanzania, Uganda, and Zambia. In 2023, seven countries (Algeria, Cote d’Ivoire, DRC, South Sudan, Tanzania, Togo, and Uganda) reported B3 genotype measles viruses. No country reported D8 viruses in 2023.

### 3.4. Verification of Measles and Rubella Elimination

By the end of 2023, a total of 18 countries (Botswana, Burundi, Cabo Verde, Cameroon, Eswatini, Gambia, Ghana, Kenya, Lesotho, Mauritius, Nigeria, Rwanda, Sao Tome e Principe, Senegal, Seychelles, Tanzania, Uganda, and Zimbabwe) had established national verification committees to support documentation of progress towards measles elimination. Sixteen of these national committees have been actively engaged in documenting the countries’ progress. WHO provided training on the verification standards to the verification committee members as well as to the national immunization and disease-control program members. In addition, WHO supported the analysis of programmatic data to be used for developing the national progress document in preparation for the verification of measles and rubella elimination. The African Regional Commission for the Verification of measles and rubella elimination (RVC) met for a third time in May 2023. However, no country in the region has attained the verification of measles or rubella elimination.

## 4. Discussion

Following the well documented declines in vaccination coverage in 2020 and 2021 related to the COVID-19 pandemic, many countries implemented efforts to recover their vaccination coverage levels in 2022 and 2023 [12,13]. The African Region witnessed little overall gain in measles vaccination (MCV1 and MCV2) coverage in 2022. Instead, the WHO UNICEF coverage estimates show that 13 countries had further decreases in MCV1 coverage in 2022 as compared to 2021 estimates [14]. Several countries, including the populous countries of Nigeria, Ethiopia, and Angola, had MCV1 coverage levels less than 60%, contributing to large numbers of children who missed measles doses in the region. 

During the years covered in this analysis, MCV1 and MCV2 coverage remained below the WHO-recommended level of 95% to attain elimination of measles. 

A common barrier to raising measles-vaccination coverage is the reluctance of health workers to open a 10-dose vial when few children are present at the vaccination post. As a result of this concern about vaccine wastage, many vaccination posts provide measles vaccines on specific days of the week only or on appointed days of the month. In addition to adopting policies and practices to allow for vaccinating every child using every possible opportunity, switching from 10-dose to 5-dose measles-containing vaccine vials can lead to reducing missed opportunities for measles vaccination [15].

The high incidence of measles in many countries in the region reflects chronic immunity gaps as a result of low coverage with two doses of MCV, as well as suboptimal implementation of SIAs (which includes delaying the implementation of time-sensitive scheduled SIAs, lack of adequate funding resources for SIAs, poor preparation, and suboptimal coverage in SIAs). Some of the challenges to attaining and sustaining high vaccination coverage include weak health systems, civil unrest, population displacement, and multiple other program priorities including disease outbreaks (e.g., vaccine-derived poliomyelitis, diphtheria, cholera, meningitis, yellow fever, etc.) [16,17].

The civil conflict which erupted in the Sudan in early 2023 leading to an influx of refugees and returnees into Chad, Ethiopia, and South Sudan is an example of an unprecedented situation that contributed to the continued high incidence of measles in the host countries [18].

In 2023, Nigeria, Guinea, and Niger reported large diphtheria outbreaks, while many countries in the African Region experienced cholera outbreaks [16,19]. These outbreaks required extensive response measures which diverted the attention and resources of national immunization program away from measles- and rubella-elimination activities. 

While there is some improvement in MCV2 coverage, the drop-out rate between the first and second doses remains high in many countries, indicating that the platform for vaccination in the second year of life has not been optimized to strengthen the routine immunization service and support measles elimination. A number of country-level studies have documented gaps in MCV2 coverage [20,21].

Addressing systemic problems with routine immunization can help countries attain measles elimination. As the contextual reasons for vaccination coverage gaps vary from country to country, countries would benefit from using tailored approaches to reach unvaccinated children. These may include improving access to vaccines through expanding fixed and/or outreach vaccination services, shifting to lower dose per measles-vaccine-containing vial formulations, conducting intensified immunization activities, and improving vaccine demand and confidence. Periodic and timely high-quality preventive SIAs remain a critical programmatic strategy to reach unvaccinated children.

The efforts to address immunization coverage gaps will benefit from addressing policy- and practice-level barriers to catching up on zero-dose or under-vaccinated children, especially those in the age group beyond two years of age who are often excluded from routine immunization services. One of these operational barriers is the fear of wasting doses in 10-dose vaccine vials. More and more countries have indicated interest to shift from 10-dose to 5-dose vials in the coming years. Using smaller dose vials ideally should create better conditions for readily opening measles-containing vaccine vials in every immunization session. In those countries that implement this shift in the doses of measles vials, there is a need to evaluate the impact of the shift from 10-dose to 5-dose vials in terms of frequency of measles vaccination service delivery, immunization coverage with MCV1 and MCV2, and in reducing missed opportunities. Data collection and monitoring systems for coverage now focus on doses delivered to children in the first year of life, and will need adaptations to capture doses of vaccines given in the second year of life or later.

WHO policy indicates that, in order to introduce rubella-containing vaccines, countries should be able to achieve 80% MCV1 coverage in routine immunization or during SIAs [22]. As of the end of 2023, 15 countries in the WHO African Region have yet to introduce rubella-containing vaccines. GAVI funding support is available for 12 of these 15 countries, provided they can demonstrate the required level of MCV1 vaccination. The 3 remaining countries (Equatorial Guinea, Gabon, and South Africa) must prioritize and self-finance the introduction of rubella vaccines, while observing the WHO rubella vaccine policy guidelines in terms of the recommended approach to introducing rubella vaccines.

Despite more than two decades of experience, SIA quality remains an issue. In 2022–2023, Gabon, Gambia, and Lesotho attained administrative SIA coverage of less than 60%. Each of these three campaigns had important contextual factors that adversely affected the coverage levels. Delayed and incomplete funding, poor level of preparations and vaccine hesitancy in the face of other concurrent events affected the coverage levels [23,24]. Overall, more than half of the SIAs in 2022 and 2023 failed to reach the 95% administrative coverage target at the national level. Only a small proportion of the countries implemented post-campaign coverage surveys to corroborate the administrative SIA coverage. Subnational level breakdown also indicates gaps which reflect the challenges with inadequate and delayed operational funding, as well as insufficient quality preparations for these mass campaigns, which are primarily organized to reach children who do not have the chance to receive routine doses of measles vaccine. 

Measles and rubella case-based surveillance continues to suffer from significant under-funding, and this further exacerbates the performance gaps. As countries make progress towards the elimination goal, there is a need to increase the sensitivity of disease surveillance, including such elements as collecting specimens from all reported febrile rash illness cases, carrying out contact tracing and detailed investigation of each chain of transmission, as well as root cause analysis of outbreaks, all of which will require committed human and funding resources. Unfortunately, the current levels of local and donor financing are not yet adequate to support the implementation of elimination standard measles-rubella surveillance. 

Attaining the elimination of measles and rubella in 80% of the countries in the region by 2030 will require significant financial investments as well as high-quality technical and political leadership to improve routine immunization and SIA coverage, as well as funding for high-quality disease surveillance. The available baseline data on circulating measles genotypes remains scant. Understanding current genotype distribution will be critical as more and more countries move closer to elimination.

Innovations to bring diagnosis and vaccination to hard to reach areas in countries of the African Region could be potentially transformative in reaching measles elimination in the region. Rapid diagnostic tests, already under field trials for measles and under development for rubella, would allow for more rapid detection and response to outbreaks [25]. The introduction of micro-array patches (MAPs) for the administration of measles and rubella vaccines would facilitate transportation of the vaccine outside of the cold chain and enable the provision of MR vaccines in areas hitherto unreached, administered by minimally trained persons, including vaccine administration on a house-to-house level [26].

Countries that have maintained high coverage and low incidence of measles may apply to be verified for the elimination of measles and/or rubella. Globally, as of April 2024, a total of 99 countries have been verified for eliminating rubella and 89 countries have been verified for eliminating measles [6]. In the African Region, countries like Botswana, Cabo Verde, Mauritius, and Seychelles have made significant progress towards eliminating measles and rubella and will need to finalize their progress documentation for review and verification by the Regional Commission for the verification of measles and rubella elimination (RVC).

This report provides valuable information on the status of measles and rubella elimination in Africa but has the following limitations. The case-based surveillance data and incidence levels do not include the data from aggregate reporting of suspected measles in countries with large outbreaks, and so represent an underestimation of the measles incidence in those countries. In countries with wide diversity in subnational level immunization coverage, the national level vaccination coverage estimates tend to mask these subnational gaps.

## 5. Conclusions

Following the COVID pandemic, the measles vaccination coverage in the African Region in 2022 remains below the targets for attaining elimination, with a large number of children who missed measles doses, and continued high incidence of measles. Countries need to provide the leadership and mobilise resources to address systemic problems with routine immunisation, and to improve the timeliness and quality of periodic SIAs to ensure reaching unvaccinated populations.

## Figures and Tables

**Table 1:** Measles vaccination first dose and second dose coverage by country, and year of rubella vaccine introduction. African Region. WHO-UNICEF coverage estimates, 2022.

Country	2022		2021		Year of Rubella Vaccine Introduction
	MCV1	MCV2	MCV1	MCV2	
Algeria	79%	71%	79%	73%	2013
Angola	37%	25%	36%	22%	2017
Benin	68%		68%		2018
Botswana	90%	77%	97%	70%	2015
Burkina Faso	88%	71%	88%	71%	2014
Burundi	89%	85%	90%	85%	2016
Cabo Verde	95%	86%	95%	86%	2009
Cameroon	65%	44%	62%	35%	2014
Central African Republic	41%		41%		
Chad	56%	2%	55%		
Comoros	86%	79%	90%	19%	2021
Congo	65%	34%	68%	31%	2018
Côte d‘Ivoire	65%	20%	68%	1%	2017
DR Congo	56%		55%		
Eq Guinea	53%	13%	53%	17%	
Eritrea	93%	85%	93%	85%	2017
Eswatini	83%	77%	80%	69%	2015
Ethiopia	56%	48%	54%	46%	
Gabon	52%		64%		
Gambia	74%	52%	79%	61%	2015
Ghana	95%	84%	94%	83%	2012
Guinea	47%	3%	47%		
Guinea-Bissau	75%	1%	63%		
Kenya	90%	56%	89%	57%	2016
Lesotho	81%	75%	90%	82%	2016
Liberia	79%	59%	58%	35%	
Madagascar	44%	32%	44%	24%	
Malawi	82%	60%	90%	74%	2016
Mali	70%	44%	70%	33%	
Mauritania	72%		63%		2017
Mauritius	98%	93%	87%	83%	1996
Mozambique	84%	70%	84%	70%	2017
Namibia	91%	79%	91%	56%	2015
Niger	65%	42%	80%	66%	
Nigeria	60%	38%	60%	38%	
Rwanda	96%	82%	87%	85%	2013
Sao Tome and Principe	77%	69%	77%	69%	2015
Senegal	80%	66%	87%	75%	2012
Seychelles	98%	98%	94%	86%	1980
Sierra Leone	90%	73%	87%	67%	2018
South Africa	86%	86%	87%	82%	
South Sudan	72%		74%		
Tanzania	86%	76%	77%	62%	2013
Togo	71%	57%	70%	50%	2017
Uganda	90%	49%	90%		2018
Zambia	90%	81%	90%	81%	2016
Zimbabwe	90%	77%	88%	76%	2014

Blank = not yet introduced.

**Table 2:** Measles supplemental immunization coverage by country. African Region, 2022–2023.

Country	Year	Type of SIAs	Age Group of Children Targeted	Number of Children Targeted	Number of Children Vaccinated	Administrative Coverage (Percent of Target)
Angola	2022	MR Follow-up	9–59 months	1,636,915	1,533,568	93.70%
Angola	2023	MR Follow-up	6–59 months	2,990,813	2,575,131	86.10%
Cameroon	2022	outbreak response	6 mo–7 years	1,179,372	1,119,550	94.90%
Cameroon	2023	MR Follow-up	9–59 months	4,416,588	3,433,312	77.70%
Central African Republic	2023	Measles follow-up	6–59 months	1,240,480	1,307,023	105.40%
Chad	2023	Measles follow-up	6–59 months	1,999,072	2,238,730	112.00%
Congo	2022	MR Follow up	6 mo–9 yrs	1,600,115	1,726,547	107.90%
DR Congo	2022	outbreak response	6–59 months	3,368,775	3,372,997	100.10%
DR Congo	2023	Measles follow-up	9–59 months	18,226,531	18,520,710	101.60%
Ethiopia	2022	outbreak response	6 mo–10 years	649,065	686,381	105.70%
Ethiopia	2022	Measles Follow-up	6–59 months	15,471,740	14,579,818	94.20%
Ethiopia	2023	Measles Follow-up	6–59 months	830,535	690,668	83.20%
Gabon	2022	Measles Follow-up	9–59 months	292,147	102,132	35.00%
Gambia	2022	MR follow-up	9–59 months	341,446	180,506	52.90%
Guinea	2022	Measles Follow-up	6–59 months	2,529,539	2,551,234	100.90%
Lesotho	2022	MR Follow-up	9–59 months	189,643	85,582	45.10%
Liberia	2022	outbreak response	9–59 months	313,817	283,579	90.40%
Madagascar	2022	Measles Follow-up	9–59 months	4,355,433	4,142,296	95.10%
Malawi	2023	MR Follow-up	9–59 months	3,180,449	2,644,144	83.10%
Mozambique	2023	MR Follow-up	9–59 months	4,811,849	5,047,614	104.90%
Namibia	2022	MR Follow-up	9–59 months	309,916	293,705	94.80%
Niger	2022	outbreak response	6–59 months	992,259	990,839	99.80%
Niger	2022	Measles follow-up	9–59 months	5,098,682	5,329,865	104.50%
Nigeria	2022	Measles follow-up	9–59 months	5,736,063	6,281,891	119.50%
Nigeria	2022	Measles follow-up	9–59 months	27,393,635	26,837,536	98.00%
Nigeria	2023	Measles follow-up	9–59 months	15,181,695	15,824,218	104.20%
S Africa	2023	Measles Follow-up	6 mo–14 yrs	17,700,872	9,593,955	54.20%
S Sudan	2023	Measles follow-up	6–59 months	2,597,415	2,383,771	91.80%
Togo	2022	MR follow-up	9–59 months	1,480,161	1,400,154	94.60%
Uganda	2022	MR follow-up	9–59 months	7,015,156	6,608,277	94.20%
Zambia	2023	outbreak response	6 mo–10 yrs	1,219,787	1,418,453	116.30%
Zimbabwe	2022	MR follow up	9–59 months	2,292,989	1,970,123	85.90%

**Table 3:** Measles and rubella case reporting by country. African Region, 2023.

Country	Total Population (In Millions)	Total Reported Measles Cases	Blood Specimens Collected	Lab Confirmed (IgM Positive) Measles	Lab Confirmed Rubella Cases	Measles Cases Confirmed by Epidemiological Linkage	Clinically Compatible Measles Cases	Total Confirmed Measles	Cases with Pending Results/Pending Classification	Measles Incidence Rate per 1,000,000 Population	Rubella Incidence Rate per 1,000,000 Population
Algeria	43.5	89	89	24	2	2	1	27	0	0.6	0.0
Angola	34.1	2960	1665	24	85	1905	18	1947	540	57.1	2.5
Benin	12.9	912	910	368	31	110	8	486	0	37.7	2.4
Botswana	2.5	1471	1434	50	62	0	1	51	0	20.5	24.9
Burkina Faso	22.9	1968	1568	596	10	968	32	1596	0	69.7	0.4
Burundi	13.1	1936	475	115	37	1460	120	1695	24	129.4	2.8
Cameroon	28.4	6812	1269	506	10	5612	56	6174	2	217.1	0.4
Cabo Verde	0.6	2	2	0	0	0	0	0	0	0.0	0.0
Central African Republic	6.4	1928	537	144	120	1388	4	1536	5	240.0	18.8
Chad	18.6	2853	2766	1130	186	87	289	1506	0	81.0	10.0
Comoros	0.8	48	48	1	2	0	0	1	10	1.2	2.4
Congo	6.1	598	399	184	5	199	9	392	0	64.7	0.8
Cote d’Ivoire	31.5	5687	5612	1286	114	75	0	1361	0	43.1	3.6
D. R. Congo	123.5	13,888	7304	4111	591	6426	125	10,662	108	86.4	4.8
Equatorial Guinea	0.9	393	264	94	4	131	13	238	36	267.9	4.5
Eritrea	3.8	105	105	18	5	0	1	19	0	5.0	1.3
Eswatini	1.2	120	120	3	1	0	0	3	1	2.6	0.9
Ethiopia	107.1	20,878	6673	2268	933	14,193	44	16,505	21	154.1	8.7
Gabon	2.5	1296	149	56	7	1145	0	1201	2	480.4	2.8
Gambia	1.7	241	239	33	13	2	2	37	0	21.2	7.5
Ghana	32.1	5588	5587	1348	92	924	41	2313	0	72.1	2.9
Guinea	13.6	533	532	28	63	177	0	205	0	15.0	4.6
Guinea Bissau	2.1	267	267	3	155	14	0	17	0	8.1	74.0
Kenya	52.3	2342	1997	580	50	247	3	830	50	15.9	1.0
Lesotho	2.1	868	868	14	9	0	1	15	0	7.2	4.3
Liberia	5.2	4525	43	43	0	4115	207	4365	0	831.6	0.0
Madagascar	29.0	2033	1775	81	389	0	9	90	269	3.1	13.4
Malawi	19.2	438	409	127	5	28	9	164	0	8.5	0.3
Mali	22.5	796	796	364	15	1	71	436	0	19.4	0.7
Mauritania	4.5	479	460	271	4	64	1	336	0	75.1	0.9
Mauritius	5.0	5	5	5	0	0	0	5	0	1.0	0.0
Mozambique	31.6	3630	3546	652	83	0	20	672	-4	21.3	2.6
Namibia	2.7	406	406	20	10	0	21	41	1	15.4	3.8
Niger	25.4	1090	1087	608	61	26	52	686	0	27.0	2.4
Nigeria	240.1	20,036	10,391	2067	2209	2209	7969	12,245	0	51.0	9.2
Rwanda	13.1	1316	1315	44	12	0	0	44	0	3.4	0.9
Saotome and Principe	0.0	0	0	0	0	0	0	0	0	0.0	0.0
Senegal	18.3	1756	1756	561	30	0	60	621	0	34.0	1.6
Seychelles	0.0	0	0	0	0	0	0	0	0	0.0	0.0
Sierra Leone	8.7	207	207	119	5	21	0	140	0	16.1	0.6
South Africa	60.6	4950	4934	884	96	0	4	888	99	14.7	1.6
South Sudan	14.6	863	808	520	45	0	31	551	37	37.7	3.1
Tanzania	61.3	4456	4208	1124	440	131	0	1255	263	20.5	7.2
Togo	8.3	883	877	547	14	52	14	613	0	73.4	1.7
Uganda	42.9	1648	1444	188	77	216	2	406	47	9.5	1.8
Zambia	18.9	1552	1549	501	50	170	30	701	53	37.0	2.6
Zimbabwe	17.0	1105	1103	17	136	0	1	18	130	1.1	8.0
**African Region**	**1213.0**	**125,957**	**77,998**	**21,727**	**6268**	**42,098**	**9269**	**73,094**	**1694**	**60.3**	**5.2**

## Data Availability

The data presented in this study are available on request from the corresponding author.
